# Impact of insecticide-treated nets and indoor residual spraying on self-reported malaria prevalence among women of reproductive age in Ghana: implication for malaria control and elimination

**DOI:** 10.1186/s12936-022-04136-3

**Published:** 2022-04-12

**Authors:** Yakubu Alhassan, Duah Dwomoh, Susan Ama Amuasi, Justice Nonvignon, Harriet Bonful, Mary Tetteh, Kofi Agyabeng, Martha Kotey, Alfred E. Yawson, Samuel Bosomprah

**Affiliations:** 1 Department of Health Policy, Planning and Management, School of Public Health, University of Ghana, Accra, Ghana; 2 Department of Biostatistics, School of Public Health, University of Ghana, Accra, Ghana; 3 Department of Physician Assistantship and Public Health, School of Medicine and Health Sciences, Central University College, Accra, Ghana; 4 Department of Epidemiology, School of Public Health, University of Ghana, Accra, Ghana; 5 Department of Community Health, University of Ghana Medical School, University of Ghana, Accra, Ghana

## Abstract

**Background:**

The Global Fund alone contributed 56% of all international financing for malaria and has invested more than US$13.5 billion in malaria treatment, prevention, and control programmes by June 2021. These investments include interventions such as mosquito nets, indoor residual spraying, and preventive treatment for children and pregnant women. However, there is paucity of studies for assessment of such investments to a reduction in malaria prevalence. This study was aimed at quantifying the impact of household access to insecticide-treated nets (ITNs) and the indoor residual spraying (IRS) on self-reported malaria prevalence among women of reproductive age in Ghana.

**Methods:**

The study analysed the 2016 Ghana Malaria Indicator Survey (MIS) data. The MIS is a nationwide survey that included women aged 15–49 years. Poisson regression model with inverse probability to treatment weighting was used to determine average treatment effect estimate of the two malaria interventions on self-reported malaria prevalence among women of reproductive age in Ghana.

**Results:**

A total sample of 4861 women interviewed from the 2016 Ghana MIS was used for analysis. The prevalence of self-reported malaria in 2016 was 34.4% (95% CI [32.4%, 36.4%]). Approximately 80.0% of women lived in households with access to ITNs [Percentage (Pr) = 79.9%, (95% CI [78.0%, 81.7%])], 12.4% (95% CI [7.5%, 19.8%]) of the households had access to IRS and 11.4% (95% CI [7.0%, 18.0%]) of the households had access to both ITNs and IRS. Household access to only ITN contributed to 7.1 percentage point (pt) reduction in the self-reported malaria among women (95% CI [− 12.0%, − 2.1%], p = 0.005) whilst IRS at the households contributed to 6.8pt reduction in malaria prevalence (95% CI [− 12.0%, − 2.1%], p = 0.005). Households with access to both ITNs and IRS contributed to a 27.1pt reduction in self-reported malaria prevalence among women (95% CI [− 12.0%, − 2.1%], p = 0.005).

**Conclusion:**

Access to both ITNs and application of IRS at the household level contributed to a significant reduction in self-reported malaria prevalence among women of reproductive age in Ghana. This finding confirms the need for integration of malaria control interventions to facilitate attainment of malaria elimination in Ghana.

**Supplementary Information:**

The online version contains supplementary material available at 10.1186/s12936-022-04136-3.

## Background

Malaria is a life-threatening disease caused by *Plasmodium* parasites transmitted through the infected bite of female *Anopheles* mosquitoes. There was an estimated 241 million malaria cases and 627,000 malaria deaths in 2020 compared to 228 million cases and 411,000 deaths in 2018 [1, 2]. The disease disproportionately affects children under the age of five years, accounting for approximately 274,000 (67%) of all malaria deaths globally in 2019. Countries in the World Health Organization (WHO) African Region have a disproportionately high share of the global malaria burden, accounting for about 94% of malaria cases and deaths. In 2019, the total funding for malaria control and elimination was estimated as USD 3 billion globally, of which about USD 900 million (31%) were contributed from governments of endemic countries [1].

One of the overarching objectives of the sustainable development goals (SDG) is to attain the highest standard of health care for everyone within all communities by preventing the occurrence of diseases [3]. Vector control has been identified as an important preventive strategy for malaria. The WHO recommends insecticide-treated nets (ITNs) and indoor residual spraying (IRS) as part of this strategy. These preventive strategies came at a huge cost with an estimated USD 3.1 billion invested in 2017 of which USD 2.2 billion were invested in the WHO African regions [2]. A total of 624 million mosquito nets were delivered from 2015 to 2017, of which 459 million (83%) ITNs were delivered in sub-Saharan Africa [2]. In 2019, it was estimated that about 46% of all people at risk of malaria in Africa were protected by an ITN, compared to 2% in 2000 [1]. However, ITN coverage has plateaued since 2016 [1]. In contrast, globally, IRS protection declined from a peak of 5% in 2010 to 2% in 2019, with decreases recorded across all WHO regions. The declines in IRS coverage are occurring as countries switch from pyrethroid insecticides to more expensive alternatives to mitigate mosquito resistance to pyrethroids [1].

In Ghana, over 13 million ITNs had been distributed as of September 2017 with about 1.5 million of those distributed in 2017 only [4]. Again, over 300,000 households were sprayed against mosquitoes protecting over 840,000 household residents through the indoor residual spraying programme [4]. Funding from the US President Malaria Initiative (PMI) over the years from an initial annual funding of USD 5 million in 2008 increased to USD 28 million in 2017 cumulating to over USD 275 million within the 10 years period. A budget of USD 26 million was made for the malaria operational plan for the 2018 fiscal year through the PMI [4]. The median cost of distribution of each ITN was estimated as USD 4.34–4.55 through mass distribution, USD 3.30 to 3.69 through school-based distribution, and USD 3.90–4.55 through health facilities [5]. The median cost of protecting an individual each year using ITNs was estimated as USD 2.20 (range: USD 0.88–9.54) whilst IRS was USD 6.70 (range: USD 2.22–12.85) [6]. Between May 2010 and October 2012, a total of 12.5 million ITNs were distributed across Ghana with an incurred cost of USD 6.51 per ITN [7]. However, there is paucity of studies quantifying the impact of these investments in terms of the distribution of ITNs and the application of IRS towards reduction of malaria prevalence in Ghana. Therefore, the aim of this study is to estimate the impact of ITNs distribution and application of IRS on malaria prevalence among women of reproductive age (15–49 years) in Ghana using self-reported malaria as a proxy for true malaria prevalence.

## Methods

### Study design and participants

Data for this study were derived from the Ghana Malaria Indicator Survey (GMIS). The GMIS is a nationally representative survey conducted by the Ghana Statistical Service from October 2016 to December 2016. For this study, only women of reproductive age 15–49 years from the survey were considered. Women who had data for all the variables were included in the analysis.

The Ghana MIS used a multi-stage cluster sampling procedure across all 10 regions of country at the time of the survey in 2016. The country was divided into 20 strata (10 regions and residential types—urban/rural). A cluster was defined as a census enumeration area (EA) comprising approximately between 300 and 500 households. In the first stage of sampling, for each stratum, clusters were selected using probability proportion to size. A total of 200 clusters were selected. In the second stage of sampling, a fixed number of 30 households were randomly selected from each selected cluster without replacement. Women aged 15–49 were interviewed from each household if available [8]. In the original survey, 5150 women were interviewed. However, due to missing responses for some of the variables, a total of 4861 women were used for this study representing 94.4% of the sampled women. The data includes information on housing, household, women characteristics, malaria prevention, and knowledge on malaria. Computer-assisted personal interviewing (CAPI) system on tablet computers and paper questionnaires were used to collected data. The Census and Survey Processing (CSPro) system was used for data editing and management by the data curators [8].

### Variable definition

#### Primary outcome

The primary outcome for this study was prevalence of self-reported malaria among women of reproductive ages 15–49 years, defined as women who reported to have experienced at least one episode of malaria within 12-months preceding the survey. That is, self-reported malaria prevalence among the women aged 15–49 was used as proxy for actual malaria RDT or microscopy positivity among the women because these tests were not performed among the women during the survey.

#### Intervention

The interventions were household access to ITNs, and application of IRS in households within 12 months prior to the survey. Households which had received both interventions were considered as integrated intervention. Household access to ITNs was defined as women who were living in households with access to at least one insecticide-treated net while household application of IRS was defined as women living in households that had been sprayed against mosquitoes within the 12 months preceding the survey.

#### Potential confounders

The study considered two main categories of confounding variables, namely, household, and individual characteristics. Household characteristics included; regions, type of residence (rural–urban), sex of household head, household size, household access to electricity, type of cooking fuel (solid or non-solid), main floor material, main wall material, roof material, source of drinking water (improved or unimproved), type of toilet facility (improved or unimproved) and household wealth category (Poor, middle and rich). Categories of the household characteristics were recoded according to the DHS reporting standards in the 2016 GMIS and 2014 Ghana Demographic Health Survey (GDHS) reports [8, 9]. Individual characteristics considered were current age of the woman, highest level of education, pregnancy status at time of survey, health insurance status, religion, exposure to malaria messages in the 6 months prior to the survey and the knowledge level of the woman on malaria issues. The knowledge level of the woman was assessed using five knowledge questions including woman’s knowledge on causes of malaria, symptoms of malaria, methods of preventing malaria, treatment of malaria and awareness that the national health insurance scheme (NHIS) of Ghana covers malaria. Women who scored 0–2 were considered to have low knowledge, those who scored 3 or 4 were considered to have moderate knowledge and those who scored five were considered to have comprehensive knowledge on malaria. The selected variables are associated with access to ITNs, IRS or malaria prevalence in literature [10–18].

### Statistical analysis

Background characteristics of women were summarized using frequencies and percentages for categorical variables whereas continuous variables were summarized using mean and standard deviation. The characteristics were summarized by intervention status to examine potential imbalance and population structure, which is an indication of potential confounding bias. Choropleth maps were used to describe prevalence of self-reported malaria among women and coverage of the two interventions by geographical location. The Rao’s Scott’s chi-square test statistic that accounts for survey design characteristics (i.e., stratification, clustering, and sampling weight) was used to assess the association between self-reported malaria prevalence and access to the two interventions and background characteristics. Self-reported malaria prevalence was calculated as the number of women who experienced at least one episode of malaria in the 12 months preceding the survey divided by the total eligible women interviewed in the survey.

A modified weighted Poisson regression model was used to estimate the impact of access to the malaria interventions on self-reported malaria prevalence among women after adjusting for the inverse probability of treatment weight (IPTW) and survey weight using the *“svy linearized”* model in Stata 16 IC (Stata Corp, College Station, TX, USA). The inverse probability of treatment weight (IPTW) for intervention *“i”* and woman *“j”* was estimated as:

$$IPTW_{ij} = \frac{i}{{pw_{ij} }} + \frac{1 - i}{{1 - pw_{ij} }}$$

where, $IPTW_{ij}$ is the inverse probability of treatment weight for intervention *i for woman j*, $pw_{ij}$ is the estimated probabitlity of woman j having access to intervention *i*, $$i\,{\mkern 1mu} {\text{is the indicator variable }}\left\{ \begin{gathered} 0{\mkern 1mu} {\mkern 1mu} {\text{if}}{\mkern 1mu}\,{\text{individual}}{\mkern 1mu}\,{\text{j}}{\mkern 1mu} \,{\text{does}}\,{\mkern 1mu} {\text{not}}{\mkern 1mu} \,{\text{have}}{\mkern 1mu} \,{\text{access}}\,{\mkern 1mu} {\text{to}}\,{\mkern 1mu} {\text{intervention}}\,{\mkern 1mu} {\mkern 1mu} i \hfill \\ 1{\mkern 1mu} \,{\text{if}}{\mkern 1mu} \,{\text{individual}}\,{\text{j}}\,{\text{have}}\,{\mkern 1mu} {\text{access}}{\mkern 1mu} \,{\text{to}}{\mkern 1mu} \,{\text{the}}\,{\mkern 1mu} {\text{intervention}}\,{\mkern 1mu} i \hfill \\ \end{gathered} \right.$$.

The final weighting variable to be used in the Poisson regression model was then adjusted as follows:

$$fw_{i} = IPTW_{i} *sw_{i}$$

where, $fw_{ij}$ is the final weighting variable for individual j and intervention *i*, $sw_{ij}$ is the sampling weight from the 2016 GMIS for individual *j* and intervention *i*.

The command “*margins, dydx (intervention_i)*” post estimation command in Stata was then used to estimate the marginal difference (impact) of access to intervention *“i”* on self-reported malaria prevalence among women after the modified weighted Poisson regression model was fitted controlling for all observed confounding variables.

As a sensitivity analysis, three different regression models, the binary logistic regression, the probit regression, and the linear regression models were also used to estimate the impact of each of the malaria interventions on self-reported malaria prevalence among women in Ghana. The 95% confidence interval was estimated for all the point prevalence estimates, prevalence ratios as well as impact estimates. All statistical analyses in this study were considered significant at an alpha level of 0.050. Stata IC version 16 (StataCorp, Texas, USA) was used for statistical analysis.

### Ethical statement

The Demographic and Health Survey (DHS) program approved and granted permission to use the data for this paper. The data was accessed from the DHS program website (http://dhsprogram.com) on 8th September 2020. The data was already de-identified and can longer be linked to any individual participant in the survey.

## Results

### Characteristics of households and women in the study

A total of 4861 women aged 15–49 years interviewed in the 2016 GMIS survey were involved in this study. A majority (53.1%) were from the urban areas of the country. The Ashanti (19.8%) and Greater Accra (18.1%) regions had the highest percentage of participants whilst the Upper East (4.0%) and Upper West (2.7%) regions had the least percentage of participants.

Approximately 36.1% of the households were headed by males. The mean (SD) age of the household head was 43.8 (13.5) years. Most (45.8%) of the women were living in household of 4–6 members. Majority of the households had access to electricity (79.5%), improved source of drinking water (87.2%), improved toilet facility (71.4%) and uses solid cooking fuel (76.6%) (Table 1).

**Table 1:** Background characteristics of women by intervention status

Variables	Number of women (%)	Access to ITNs		Household Sprayed (IRS)		Combination of interventions				
						None	IRS only	ITNs only	Both IRS & ITNs	P-value
		%	P-value	%	P-value	%	%	%	%	
Total	**4861 (100%)**	79.91		12.43		19.07	1.02	68.5	11.41	
Region of residence			< 0.001		< 0.001					< 0.001
Western	398 (8.19)	5.99		1.15		2.06	0.14	4.98	1.01	
Central	511 (10.51)	9.42		0.78		1.09	0.00	8.64	0.78	
Greater Accra	882 (18.15)	12.86		0.29		5.29	0.00	12.57	0.29	
Volta	401 (8.24)	6.79		0.00		1.46	0.00	6.79	0.00	
Eastern	451 (9.28)	7.09		0.00		2.19	0.00	7.09	0.00	
Ashanti	964 (19.84)	14.87		2.00		4.59	0.37	13.25	1.62	
Brong Ahafo	420 (8.64)	7.27		0.23		1.35	0.01	7.05	0.22	
Northern	509 (10.48)	9.21		4.47		0.93	0.34	5.08	4.13	
Upper East	192 (3.95)	3.86		1.01		0.08	0.01	2.85	1.00	
Upper West	133 (2.73)	2.56		2.50		0.03	0.15	0.20	2.35	
Place of residence			< 0.001		0.005					< 0.001
Urban	2579 (53.06)	39.16		3.27		13.62	0.29	36.17	2.99	
Rural	2282 (46.94)	40.75		9.15		5.45	0.73	32.33	8.42	
Household characteristics										
Household size			< 0.001		< 0.001					< 0.001
< 4 members	1448 (29.79)	21.79		2.21		7.60	0.40	19.98	1.81	
4–6 members	2225 (45.78)	37.29		5.28		8.22	0.27	32.28	5.01	
7–9 members	904 (18.6)	15.73		3.61		2.52	0.35	12.46	3.27	
10+ members	284 (5.83)	5.11		1.32		0.73	0.00	3.78	1.32	
Sex of household head			< 0.001		0.007					< 0.001
Male	3108 (63.93)	53.06		9.87		10.2	0.67	43.86	9.20	
Female	1753 (36.07)	26.85		2.56		8.86	0.36	24.64	2.21	
Age of household head (mean ± SD)	43.75 ± 13.46		0.041		0.183					0.054
< 30	668 (13.74)	10.14		1.20		3.55	0.05	8.99	1.16	
30–49	2746 (56.49)	45.84		7.60		9.92	0.73	38.97	6.87	
50–69	1206 (24.81)	19.75		3.07		4.84	0.22	16.90	2.85	
> 69	241 (4.961)	4.17		0.55		0.76	0.02	3.64	0.53	
Wealth index			< 0.001		0.011					< 0.001
Poor	1705 (35.08)	30.82		6.48		4.09	0.17	24.51	6.31	
Middle	1000 (20.56)	16.60		2.82		3.66	0.30	14.09	2.52	
Rich	2156 (44.36)	32.48		3.13		11.32	0.56	29.90	2.58	
Source of water			0.004		0.857					0.324
Improved water source	4239 (87.22)	68.87		10.95		17.37	0.97	58.89	9.98	
Unimproved water source	621 (12.78)	11.03		1.48		1.70	0.05	9.61	1.42	
Toilet facility			< 0.001		0.007					0.002
Improved toilet facility	3468 (71.35)	55.38		6.73		15.34	0.63	49.28	6.10	
Unimproved toilet facility	1393 (28.65)	24.52		5.70		3.73	0.39	19.22	5.31	
Access to electricity			< 0.001		0.524					0.045
No	996 (20.48)	18.29		3.04		2.06	0.13	15.39	2.91	
Yes	3865 (79.52)	61.61		9.39		17.01	0.89	53.11	8.50	
Main floor materials			0.058		0.076					0.087
Ceramic/tiles/carpet	1165 (23.97)	18.20		2.05		5.51	0.26	16.41	1.79	
Cement	3051 (62.78)	50.66		8.16		11.55	0.57	43.07	7.59	
Sand/earth/wooden planks	644 (13.25)	11.05		2.22		2.01	0.19	9.02	2.02	
Main wall materials			< 0.001		0.004					< 0.001
Cement/bricks	3143 (64.66)	48.77		4.97		15.27	0.61	44.41	4.36	
Others (clay, woods etc.,)	1718 (35.34)	31.13		7.46		3.80	0.41	24.09	7.05	
Main roof materials			0.002		< 0.001					< 0.001
Asbestos/shingles/concrete	871 (17.92)	12.90		0.26		4.96	0.06	12.70	0.21	
Zinc/aluminium	3810 (78.37)	63.75		11.38		13.68	0.95	53.32	10.43	
Thatch/palm leaves/wood	180 (3.709)	3.25		0.78		0.44	0.02	2.49	0.77	
Cooking fuel			< 0.001		0.064					0.003
Non-solid (LPG, electricity)	1137 (23.39)	16.48		1.55		6.64	0.27	15.21	1.27	
Solid (charcoal, woods, etc.)	3724 (76.61)	63.43		10.88		12.43	0.75	53.29	10.13	
Women characteristics										
Woman’s age	29.80 ± 9.51		0.91		0.439					0.7016
15–19	854 (17.56)	14.20		2.55		3.08	0.28	11.93	2.27	
20–29	1610 (33.12)	26.36		3.88		6.47	0.29	22.77	3.59	
30–39	1458 (29.99)	23.88		3.66		5.86	0.25	20.47	3.41	
40–49	939 (19.33)	15.46		2.33		3.66	0.21	13.34	2.13	
Woman’s education			0.009		< 0.001					< 0.001
No education	955 (19.65)	16.46		4.62		2.75	0.44	12.28	4.18	
Primary	832 (17.12)	13.91		2.23		2.92	0.28	11.96	1.95	
Secondary	2719 (55.94)	44.18		4.80		11.46	0.30	39.68	4.50	
Higher/tertiary	355 (7.29)	5.35		0.77		1.94	0.00	4.58	0.77	
Number of births			0.007		0.204					0.076
None	1391 (28.61)	21.81		3.12		6.44	0.36	19.05	2.76	
1–2 births	1406 (28.92)	22.89		3.39		5.75	0.28	19.77	3.12	
3–4 births	1095 (22.52)	18.56		2.73		3.85	0.10	15.93	2.63	
> 4 births	970 (19.96)	16.65		3.18		3.02	0.29	13.75	2.89	
Woman’s currently pregnant			0.079		0.134					0.164
No/unsure	4511 (92.8)	73.84		11.28		18.05	0.90	63.47	10.38	
Yes	350 (7.2)	6.06		1.15		1.02	0.12	5.03	1.03	
Covered by health insurance			0.135		< 0.001					0.003
No	2011 (41.38)	32.44		3.98		8.71	0.22	28.68	3.76	
Yes	2850 (58.62)	47.47		8.45		10.36	0.80	39.82	7.65	
Woman’s religion			0.277		0.005					0.004
Christians	3760 (77.35)	61.74		6.48		15.12	0.48	55.74	6.00	
Islam	946 (19.46)	15.83		5.71		3.13	0.49	10.61	5.22	
Tradition/no religion/others	155 (3.19)	2.33		0.24		0.81	0.05	2.15	0.18	
Knowledge of malaria			< 0.001		0.1254					0.016
Low knowledge	81 (1.66)	1.25		0.15		0.38	0.04	1.13	0.11	
Moderate knowledge	1464 (30.11)	22.90		3.10		6.89	0.33	20.12	2.77	
Comprehensive knowledge	3316 (68.22)	55.77		9.18		11.80	0.66	47.24	8.52	
Exposure to malaria messages in the past 6 months			0.942		0.167					0.194
Not exposed	2633 (54.17)	43.31		7.67		10.07	0.79	36.43	6.88	
Exposed	2228 (45.83)	36.59		4.76		9.00	0.24	32.07	4.52	

ITN, insecticide treated net; IRS, indoor residual spraying; CI, confidence interval

All percentages are column percentages P-values are from the Rao Scott’s chi-square tests

The mean (SD) age of the women was 29.8 (9.5) years. In most (55.9%) cases, the women had up to secondary level of education while few of them had beyond secondary education. Christianity was the most (77.4%) affiliated religion among the women. Over a quarter (28.6%) of the women had never given birth, another 28.9% had given birth once or twice whilst a fifth (20.0%) had given birth for more than four times. About seven in every ten women sampled (68.2%) had a comprehensive knowledge of malaria. However, more than half (54.2%) of the women had been exposed to malaria messages in the past 6 months (Table 1).

### Prevalence of self-reported malaria and access to malaria interventions

**Fig. 1:**
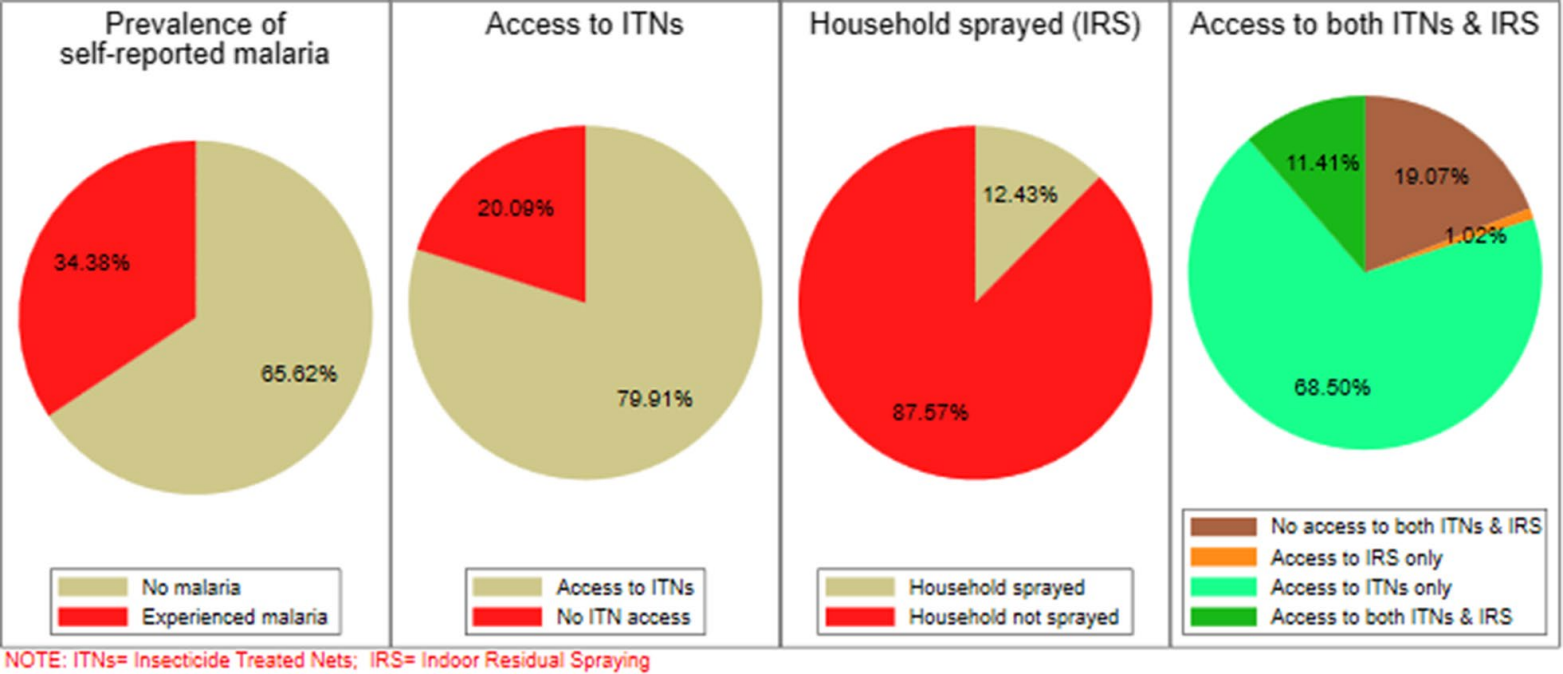
Prevalence of self-reported malaria and access to malaria interventions among women aged 15–49 years in Ghana

The prevalence of self-reported malaria in the last 12 months prior to the survey was 34.4% (95% CI 32.4–36.4%). The percentage of women with access to ITNs was 79.9% (95% CI 78.0–81.7%) whereas women living in household sprayed against mosquitoes (IRS) was 12.4% (95% CI 7.5–19.8%). Access to only IRS was 1.0% (95% CI 17.1–21.2%), only ITNs was 68.5% (95% CI 62.9–73.6%) and both IRS and ITNs was 11.4% (95% CI 7.0–18.0%) (Fig. 1).

Access to ITNs was significantly associated with region (p < 0.001), area of residence (p < 0.001), household size (p < 0.001), Sex of household head (p < 0.001), age of household head (p = 0.041), household wealth index category (p < 0.001), source of drinking water (p = 0.004), type of toilet facility (p < 0.001), access to electricity (p < 0.001), type of cooking fuel (p < 0.001) and housing characteristics such as main wall material (p < 0.001) and main roof material (p < 0.001). In addition, women characteristics such as education (p = 0.009), number of births (p = 0.007) and knowledge of malaria (p < 0.001) were also associated with access to ITNs (Table 1).

Household characteristics associated with access to IRS included region (p < 0.001), place of residence (p = 0.005), household size (p < 0.001), sex of household head (p = 0.007), wealth index (p = 0.011), type of toilet facility (p = 0.007), main wall material (p = 0.004) and main roof material (p < 0.001). The women characteristics associated with access to IRS among the women included education (p < 0.001), health insurance status (p < 0.001) and religion (p = 0.005) (Table 1).

### Regional distribution of self-reported malaria prevalence and access to malaria interventions

**Fig. 2:**
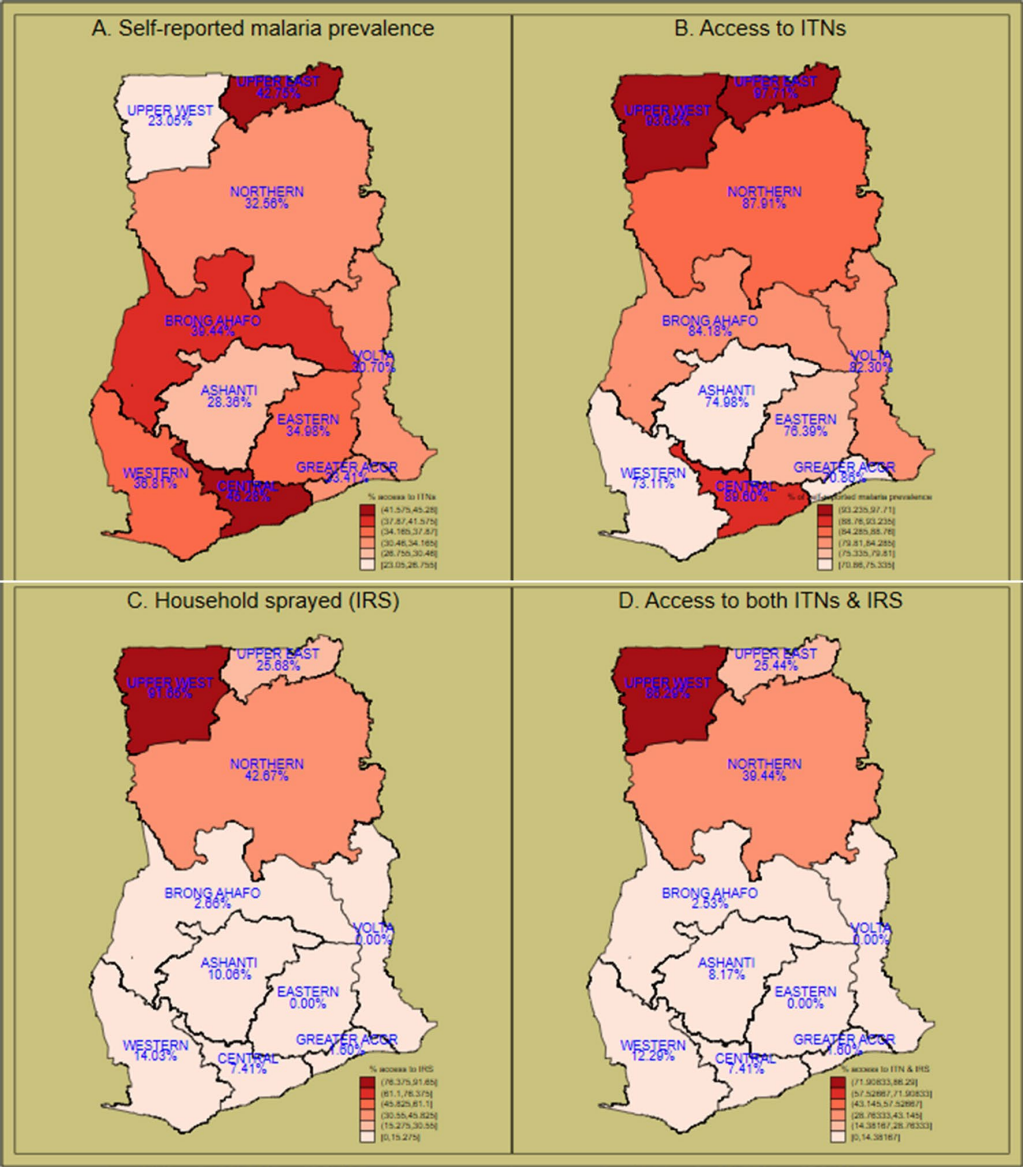
Prevalence of self-reported malaria and access to malaria interventions among women by regions

The Upper East (42.8%) and the Central (45.3%) recorded the highest self-reported malaria prevalence whilst the Upper West (23.1%) and Ashanti (28.4%) recorded the least prevalence. Access to ITNs was highest in the Upper West (93.6%) and the Upper East (97.7%) regions whilst Greater Accra (70.9%), Western (73.1%) and Ashanti (75.0%) recorded the least percentage access. The percentage of women with access to IRS was highest in the Upper West region (91.7%) followed by the Northern region with 42.7% and Upper East with 25.6% whilst the rest of the southern regions recorded less than 15% each with the Volta and Eastern regions recording 0%. Access to both ITNs and IRS was highest in the three northern regions, Upper West (86.3%), Northern (39.4%) and Upper East (25.4%) (Fig. 2).

### Prevalence of self-reported malaria among women 12 month before the survey by access to malaria interventions

Prevalence of self-reported malaria among women with access to ITNs was 33.3% (95% CI 31.2–35.4%) which was significantly lower compared to the 38.7% (95% CI 33.9–43.7%) among women with no access to ITNs (χ^2^ = 4.32, p = 0.039). Self-reported malaria did not significantly vary between women with access to IRS (32.3%, 95% CI 28.1–36.9%) compared to women with no access to IRS (34.7%, 95% CI 32.6–36.8%) (χ^2^ = 0.91, p = 0.342). Also, self-reported malaria among the women did not significantly differ across the combination of access to the two malaria interventions (χ^2^ = 1.65, p = 0.188) (Table 2).

**Table 2:** Prevalence of self-reported malaria among women 12 month before the survey by access to malaria interventions

	Experienced malaria in the past 12 months		Rao Scott's Chi-square	P-value
	No	Yes		
	% [95% CI]	% [95% CI]		
	65.62 [63.63, 67.57]	34.38 [32.43, 36.37]		
Interventions				
Insecticide treated nets (ITNs)			4.32	0.039
No access to ITNS	61.33 [56.31, 66.11]	38.67 [33.89, 43.69]		
Access to ITNs	66.71 [64.59, 68.76]	33.29 [31.24, 35.41]		
Indoor residual spraying (IRS)			0.91	0.342
Household not sprayed	65.34 [63.18, 67.43]	34.66 [32.57, 36.82]		
Household sprayed	67.63 [63.07, 71.87]	32.37 [28.13, 36.93]		
Both ITNs and IRS			1.65	0.188
No access to both ITNs & IRS	61.31 [56.44, 65.96]	38.69 [34.04, 43.56]		
Access to only IRS	61.65 [39.00, 80.17]	38.35 [19.83, 61.00]		
Access to only ITNs	66.46 [64.27, 68.59]	33.54 [31.41, 35.73]		
Access to both ITNs & IRS	68.16 [62.41, 73.42]	31.84 [26.58, 37.59]		
Region of residence			4.38	< 0.001
Western	63.19 [57.76,68.30]	36.81 [31.70,42.24]		
Central	54.72 [48.15,61.13]	45.28 [38.87,51.85]		
Greater Accra	66.59 [61.80,71.07]	33.41 [28.93,38.20]		
Volta	69.30 [64.06,74.08]	30.70 [25.92,35.94]		
Eastern	65.02 [59.91,69.80]	34.98 [30.20,40.09]		
Ashanti	71.64 [65.87,76.78]	28.36 [23.22,34.13]		
Brong Ahafo	60.56 [53.22,67.44]	39.44 [32.56,46.78]		
Northern	67.44 [62.39,72.11]	32.56 [27.89,37.61]		
Upper East	57.25 [50.71,63.54]	42.75 [36.46,49.29]		
Upper West	76.95 [70.86,82.09]	23.05 [17.91,29.14]		
Place of residence			0.00	0.970
Urban	65.59 [62.88,68.20]	34.41 [31.80,37.12]		
Rural	65.66 [62.68,68.53]	34.34 [31.47,37.32]		
Household characteristics				
Household size			0.89	0.440
< 4 members	64.02 [60.42,67.46]	35.98 [32.54,39.58]		
4–6 members	65.52 [62.53,68.39]	34.48 [31.61,37.47]		
7–9 members	67.45 [63.50,71.17]	32.55 [28.83,36.50]		
10+ members	68.81 [62.58,74.43]	31.19 [25.57,37.42]		
Sex of household head			0.63	0.430
Male	66.14 [63.87,68.33]	33.86 [31.67,36.13]		
Female	64.71 [61.47,67.83]	35.29 [32.17,38.53]		
Age of household head (mean ± SD)			0.44	0.702
< 30	64.54 [59.04,69.69]	35.46 [30.31,40.96]		
30–49	65.47 [62.57,68.27]	34.53 [31.73,37.43]		
50–69	65.69 [62.45,68.79]	34.31 [31.21,37.55]		
> 69	69.99 [62.22,76.76]	30.01 [23.24,37.78]		
Wealth index			2.26	0.106
Poor	67.68 [64.29,70.88]	32.32 [29.12,35.71]		
Middle	61.91 [57.74,65.90]	38.09 [34.10,42.26]		
Rich	65.73 [62.61,68.71]	34.27 [31.29,37.39]		
Source of water			12.57	< 0.001
Improved water source	64.59 [62.48,66.64]	35.41 [33.36,37.52]		
Unimproved water source	72.70 [68.54,76.49]	27.30 [23.51,31.46]		
Toilet facility			0.14	0.713
Improved toilet facility	65.39 [62.95,67.74]	34.61 [32.26,37.05]		
Unimproved toilet facility	66.22 [62.45,69.80]	33.78 [30.20,37.55]		
Access to electricity			1.98	0.161
No	68.52 [63.69,72.97]	31.48 [27.03,36.31]		
Yes	64.88 [62.75,66.95]	35.12 [33.05,37.25]		
Main floor materials			2.50	0.083
Ceramic/tiles/carpet	62.18 [58.53,65.70]	37.82 [34.30,41.47]		
Cement	66.35 [63.91,68.71]	33.65 [31.29,36.09]		
Sand/earth/wooden planks	68.41 [63.05,73.33]	31.59 [26.67,36.95]		
Main wall materials			0.49	0.485
Cement/bricks	65.20 [62.70,67.61]	34.80 [32.39,37.30]		
Others (clay, woods etc.,)	66.40 [63.63,69.06]	33.60 [30.94,36.37]		
Main roof materials			0.50	0.587
Asbestos/shingles/concrete	66.37 [61.43,70.97]	33.63 [29.03,38.57]		
Zinc/aluminium	65.27 [63.05,67.43]	34.73 [32.57,36.95]		
Thatch/palm leaves/wood	69.50 [62.36,75.81]	30.50 [24.19,37.64]		
Cooking fuel			0.61	0.435
Non-solid (LPG, electricity)	64.47 [60.94,67.84]	35.53 [32.16,39.06]		
Solid (charcoal, woods, etc.)	65.98 [63.73,68.15]	34.02 [31.85,36.27]		
Women characteristics				
Woman’s age			8.14	< 0.001
15–19	74.63 [71.32,77.68]	25.37 [22.32,28.68]		
20–29	64.45 [60.87,67.87]	35.55 [32.13,39.13]		
30–39	62.91 [59.45,66.24]	37.09 [33.76,40.55]		
40–49	63.67 [59.66,67.50]	36.33 [32.50,40.34]		
Woman’s education			0.68	0.537
No education	68.35 [63.02,73.24]	31.65 [26.76,36.98]		
Primary	64.97 [61.44,68.35]	35.03 [31.65,38.56]		
Secondary	64.99 [62.40,67.50]	35.01 [32.50,37.60]		
Higher/tertiary	64.66 [57.68,71.07]	35.34 [28.93,42.32]		
Number of births			2.24	0.087
None	68.29 [65.33,71.12]	31.71 [28.88,34.67]		
1–2 births	64.80 [61.32,68.13]	35.20 [31.87,38.68]		
3–4 births	66.40 [62.68,69.92]	33.60 [30.08,37.32]		
> 4 births	62.12 [57.80,66.25]	37.88 [33.75,42.20]		
Woman’s currently pregnant			0.07	0.787
No/unsure	65.70 [63.61,67.73]	34.30 [32.27,36.39]		
Yes	64.67 [57.16,71.51]	35.33 [28.49,42.84]		
Covered by health insurance			0.23	0.630
No	66.18 [62.98,69.24]	33.82 [30.76,37.02]		
Yes	65.23 [62.77,67.62]	34.77 [32.38,37.23]		
Woman’s religion			1.97	0.143
Christians	65.61 [63.28,67.87]	34.39 [32.13,36.72]		
Islam	64.35 [60.67,67.87]	35.65 [32.13,39.33]		
Tradition/no religion/others	73.63 [65.73,80.25]	26.37 [19.75,34.27]		
Knowledge of malaria			7.03	0.002
Low knowledge	88.59 [80.26,93.69]	11.41 [6.31,19.74]		
Moderate knowledge	66.17 [62.50,69.65]	33.83 [30.35,37.50]		
Comprehensive knowledge	64.82 [62.50,67.08]	35.18 [32.92,37.50]		
Exposure to malaria messages in the past 6 months			34.07	< 0.001
Not exposed	70.47 [67.96,72.87]	29.53 [27.13,32.04]		
Exposed	59.89 [57.03,62.69]	40.11 [37.31,42.97]		

ITN, insecticide treated net; IRS, indoor residual spraying; CI, confidence interval

All percentages are row percentages

### Factors associated with self-reported malaria prevalence among women in the past 12 months

Prevalence of self-reported malaria was significantly associated with the region of residence of the women (χ^2^ = 4.38, p < 0.001). Self-reported malaria prevalence was highest among women with access to improved water sources (35.4%, 95% CI 33.4–37.5%) compared to the 27.3% (95% CI 23.5–31.5%) prevalence among women with access to unimproved water sources (χ^2^ = 12.57, p < 0.001). Also, self-reported malaria prevalence was lowest among women in the age range 15–19 years (25.4%, 95% CI 22.3–28.7%) compared to women in the age groups 20–29 years (35.6%, 95% CI 31.7–38.5%), 30–39 years (37.1%, 95% CI 33.8–40.6%) and those aged 40–49 years (36.3%, 95% CI 32.5–40.3%). The age group of women was significantly associated with self-reported malaria prevalence (χ^2^ = 8.14, p < 0.001). Self-reported malaria was lowest among women with low knowledge on malaria (11.4%, 95% CI 6.3–19.7%) compared to women with moderate (33.8%, 95% CI 30.4–37.5%) or comprehensive (35.2%, 95% CI 32.9–37.5%) knowledge (χ^2^ = 7.03, p = 0.002). Also, self-reported malaria prevalence was highest among women exposed to malaria messages (40.1%, 95% CI 37.3–43.0%) compared to women not exposed to malaria messages (29.5%, 95% CI 27.1–32.0%) (χ^2^ = 34.07, p < 0.001) (Table 2).

### The impact of household access to ITNs and application of IRS on self-reported malaria prevalence

Women living in households with access to ITNs recorded a 7.05% significant absolute reduction in self-reported malaria prevalence [ATE − 7.05%, 95% CI (− 11.96%, − 2.14%), p = 0.005]. Women living in households with access IRS had a 6.81% significant reduction in self-reported malaria prevalence [ATE: − 6.81%, 95% CI (− 13.06%, − 0.55%), p = 0.033] (Table 3).

**Table 3:** The impact of malaria control interventions on self-reported malaria prevalence among women in the past 12 months

Intervention arm vs. Non-intervention arm	Impact estimates of the malaria interventions on self-reported malaria prevalence among women aged 15–49 years							
	Poisson regression model		Sensitivity analysis					
			Binary logistic regression model		Probit regression model		Linear regression model	
	ATE [95% CI]	P-value	ATE [95% CI]	P-value	ATE [95% CI]	P-value	ATE [95% CI]	P-value
Access to ITNs vs. No access to ITNs	− 7.05 [− 11.96, − 2.14]	0.005	− 7.88 [− 13.14, − 2.62]	0.004	− 7.16 [− 12.26, − 2.07]	0.006	− 7.39 [− 12.60, − 2.17]	0.006
Household sprayed (IRS) vs. Household not sprayed	− 6.81 [− 13.06, − 0.55]	0.033	− 6.36 [− 13.03, 0.32]	0.062	− 7.34 [− 14.10, − 0.58]	0.033	− 5.99 [− 12.20, 0.23]	0.059
Access to ITNs & IRS vs. Access to ITNs only	− 6.88 [− 14.69, 0.93]	0.084	− 6.83 [− 14.61, 0.94]	0.085	− 7.68 [− 15.63, 0.28]	0.059	− 6.41 [− 13.86, 1.03]	0.091
Access to ITNs & IRS vs. Access to IRS only	− 4.70 [− 9.76, 0.37]	0.068	− 4.12 [− 8.15, − 0.09]	0.045	− 3.25 [− 7.09, 0.59]	0.095	− 19.08 [− 38.97, 0.80]	0.060
Access to ITNs & IRS vs. No access to ITNs & IRS	− 27.09 [− 34.94, − 19.25]	< 0.001	− 27.99 [− 35.58, − 20.41]	< 0.001	− 28.66 [− 36.33, − 21.00]	< 0.001	− 27.12 [− 35.62, − 18.63]	< 0.001

Household characteristics (region, residence, household size, sex of household head, age of household head, household wealth quintile, household water source, toilet facility, access to electricity, main floor materials, main wall materials, main roof material and type of cooking fuel) and women individual characteristics (age of woman, highest education, religion, number of children, currently pregnant, covered by health insurance, knowledge on malaria and exposure to malaria message in the past 6 months) were controlled for

ATE, average treatment effect. Percentage difference in malaria prevalence (Intervention − No Intervention); CI, confidence interval; ITNs, insecticide treated nets; IRS, indoor residual spraying

Compared to those with access to only ITNs, access to both ITNs and IRS did not show significant reduction in self-reported malaria prevalence among the women in any of the four regression models. Also, compared to those with access to IRS only, access to both ITNs and IRS did not show significant reduction in malaria prevalence in the final model (Table 3).

Compared to those with no access to both ITNs and IRS, access to both ITNs and IRS contributed a 27.09% significant absolute reduction in self-reported malaria prevalence among the women [ATE: − 27.09, 95% CI (− 34.94%, − 19.25%), p < 0.001] (Table 3).

### Subgroup analysis of the impact of household access to ITNs and application of IRS on self-reported malaria prevalence

**Fig. 3:**
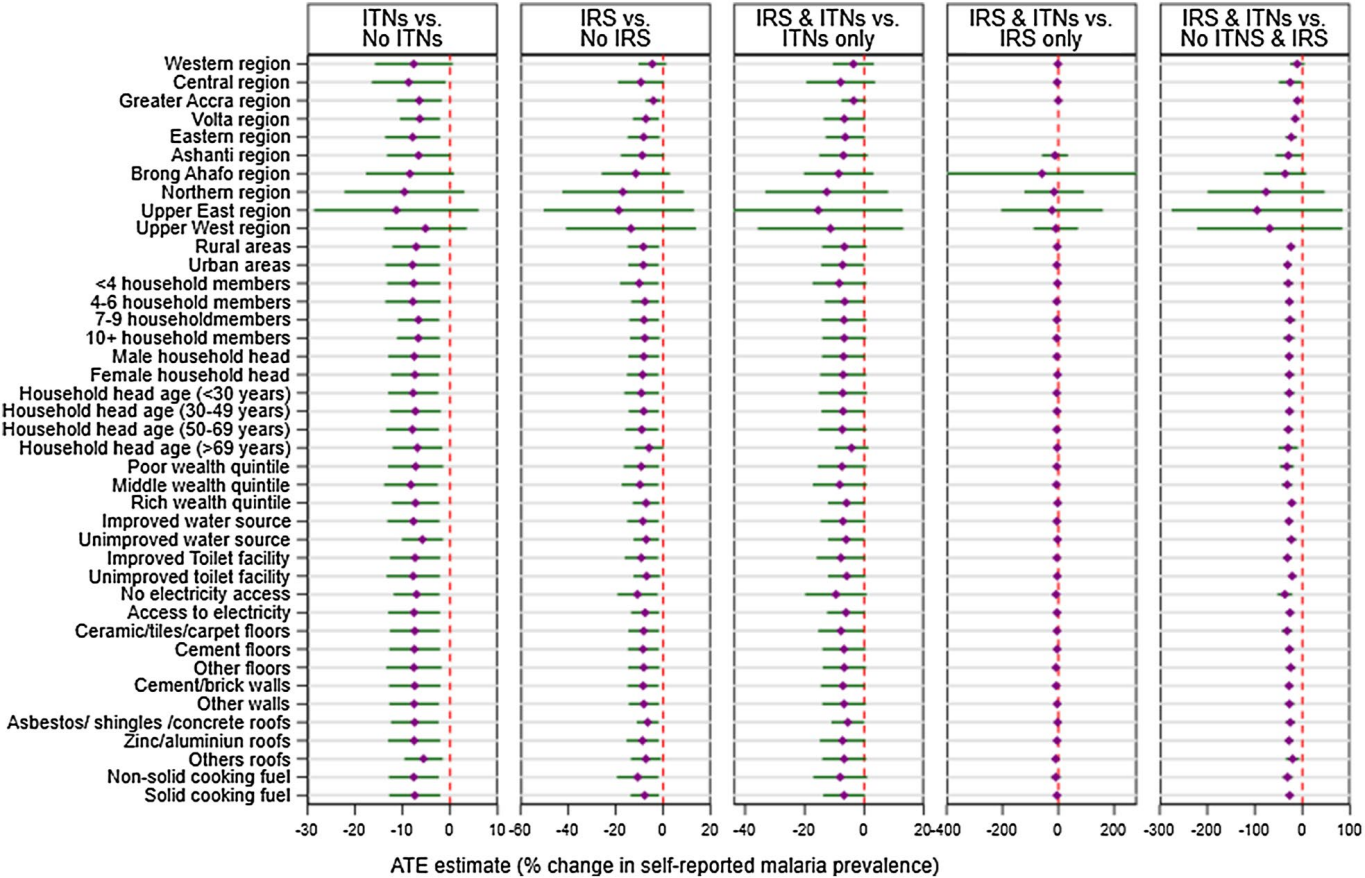
Impact of access to malaria intervention on self-reported malaria prevalence among women by household characteristics

**Fig. 4:**
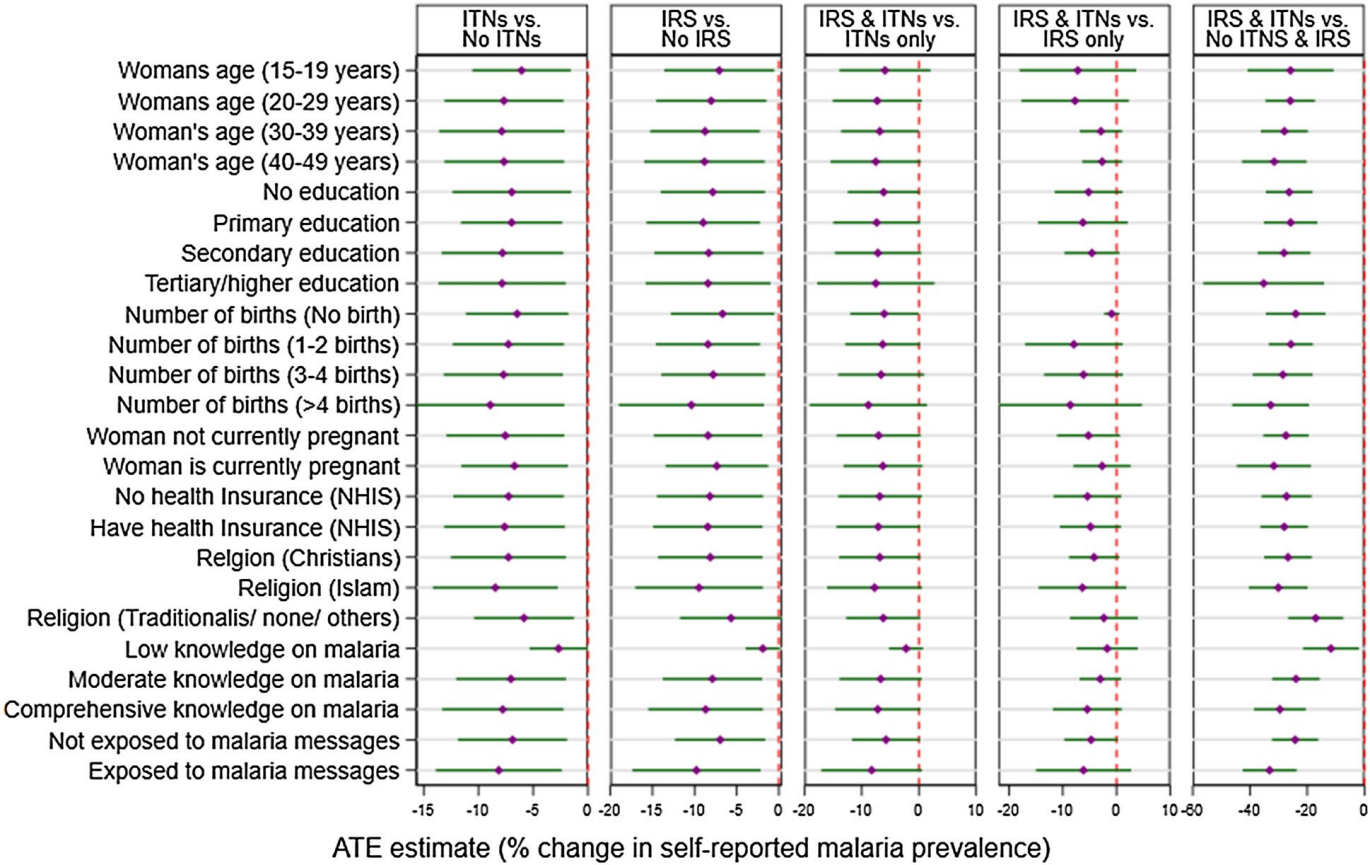
Impact of access to malaria intervention on self-reported malaria prevalence among women by women characteristics

Access to ITNs contributed to a significant reduction in self-reported malaria prevalence in the central (ATE: − 8.71%, 95% CI [− 16.49, − 0.92], p = 0.029), Greater Accra (ATE: − 6.49%, 95% CI [− 11.14, − 1.79], p = 0.007), Volta (ATE: − 6.33%, 95% CI [− 10.51, − 2.15], p = 0.003), and the Eastern (ATE: − 7.89%, 95% CI [− 13.66, − 2.07], p = 0.008) regions. Also, access to ITNs contributed over 7% significant reduction in both the urban (ATE: − 7.14%, 95% CI: [− 12.13, − 2.14], p = 0.005) and the rural areas (ATE: − 7.88%, 95% CI [− 13.60, − 2.16], p = 0.007). All other subgroups of the household characteristics and women individual characteristics also showed varying significant reduction in self-reported malaria prevalence among women with access to ITNs ranging from over 2% reduction among women with low knowledge on malaria (ATE: − 2.67%, 95% CI: [− 5.53, − 0.02], p = 0.048) to over 8% reduction among women with more than 4 births (ATE: − 8.92%, 95% CI [− 15.69, − 2.15], p = 0.010) (Figs. 3 and 4, and Additional file 1: Table S1).

Access to IRS contributed to significant reduction in self-reported malaria prevalence in the Greater Accra (ATE: − 4.10%, 95% CI [− 7.37, − 0.83], p = 0.014), Volta (ATE: − 7.29%, 95% CI [− 12.78, − 1.81], p = 0.009), and the Eastern (ATE: − 8.20%, 95% CI [− 14.89, − 1.52], p = 0.016) regions. Also, access to IRS contributed to over 8% significant reduction in both the urban areas (ATE: − 8.35%, 95% CI [− 14.96, − 1.75], p = 0.013) and the rural areas (ATE: − 8.30%, 95% CI [− 14.64, − 1.96], p = 0.011). Results of the impact of IRS on self-reported malaria reduction among women by both household characteristics and women individual characteristics are shown in Figs. 3 and 4, and Additional file 1: Table S1.

Access to both ITNs and IRS contributed to significant reduction in self-reported malaria prevalence in the central (ATE: − 25.77%, 95% CI [− 49.52, − 2.01], p = 0.034), Greater Accra (ATE: − 10.84%, 95% CI [− 18.40, − 3.28], p = 0.005), Volta (ATE: − 15.04%, 95% CI [− 22.18, − 7.90], p < 0.001), the Eastern (ATE: − 23.54%, 95% CI [− 35.43, − 11.65], p < 0.001) and the Ashanti (ATE: − 29.34%, 95% CI [− 56.51, − 2.18], p = 0.034) regions. Also, access to both ITNs and IRS contributed to significant reduction in both the urban (ATE: − 24.22%, 95% CI [− 32.65, − 15.78], p < 0.001) and the rural areas (ATE: − 30.94%, 95% CI [− 39.66, − 22.22], p < 0.001). All the other subgroups of the household characteristics and women individual characteristics also showed varying significant reduction in self-reported malaria prevalence among women with access to both ITNs and IRS ranging from over 11% among women with low knowledge on malaria (ATE: − 11.69, 95% CI [− 21.42, − 1.96], p = 0.019) to over 36% reduction among women living in household with no access to electricity (ATE: − 36.96, 95% CI [− 52.52, − 21.40], p < 0.001)(Figs. 3 and 4, and Additional file 1: Table S1).

## Discussion

The package of vector controlled preventive strategy for malaria contributed to significant reduction in self-reported malaria prevalence among women of reproductive age in Ghana. Access to both ITNs and IRS among women recorded a 27% reduction in self-reported malaria prevalence. This finding is consistent with the results from a randomized controlled trial which showed evidence of significant reduction in malaria RDT positivity among IRS users compared to non-IRS users in a high malaria endemic but high standard ITNs access area in Mozambique [19]. In Northern Tanzania, the combination of ITNs and IRS recorded a significant reduction in the *Anopheles* density and entomological inoculation rates [20]. The finding on an integrated vector-controlled preventive strategy for malaria is further supported by a community-based survey conducted in Nyanza province in Western Kenya which found that the combination of indoor residual spraying and insecticide-treated nets provided added protection against malaria compared with insecticide-treated nets alone [21].

There was a 7% reduction in reported malaria prevalence among women with access to ITNs with a 95% confidence reduction of 2–12% from this study. Comparable results were reported from a trend of malaria cases in health sentinel sites in Papua New Guinea which also recorded a reduction in malaria cases because of the repeated distribution of long-lasting insecticidal nets [22]. In the Tombel Health District, Southwest region of Cameroon, the distribution of ITNs recorded a short-lived reduction of malaria cases from three health facilities in 2012 (22.7%) following the distribution of ITNs compared to post-distribution cases in 2010 (26.7%) and 2011 (30.7%). However, the cases recorded an increase to 29.5% in 2013 from 22.7% in 2012 [23]. Comparable results were recorded for IRS alone. For example, compared to no IRS, we found that women living in households sprayed against mosquitoes or treated with indoor residual insecticide recorded a 6.8% absolute reduction in self-reported malaria prevalence. This was also consistent with findings from a district-level observational study in the northern region of Ghana in which there was a 39%, 26% and 58% relative reduction in confirmed malaria cases in 2015, 2016 and 2017 respectively among IRS campaigned districts compared to non-IRS campaigned districts [24]. In another study in the Bunkpurugu-yunyoo district in northern region of Ghana, there was an estimated 5% marginal decline in asexual parasitaemia prevalence among children from 52% in November 2010 to 48% in October 2012 during a high transmission season after application of alpha-cypermethrin IRS between the two periods. There was a further decline in malaria parasitaemia prevalence from 48% in October 2012 to 20.6% in October 2013 after pirimiphos-methyl IRS application [25].

This study estimated that 34% of women had malaria episode 12 months before the survey with a 95% confidence interval estimate of 32–36%. The prevalence of self-reported malaria episode among women living in a household with access to ITNs (33%) was significantly lower than women living in households with no access to ITNs (39%). Similarly, the prevalence of self-reported malaria among women living in a household that had been sprayed against mosquitoes was 32% compared to 35% in household that had not been sprayed. Unimproved toilet facilities and poor sanitary conditions mostly includes open spaces where dirty water is stagnated. These stagnated dirty water bodies are the optimal environment for breeding the anopheles’ mosquitoes which is the main vector for transmitting malaria in Ghana. Unimproved toilet facilities and sanitary condition, therefore, directly lead to increase in community spread of malaria. Efforts towards the provision of improved toilet facilities and sanitations in households and communities should be strengthen.

## Study limitations

This study had several important limitations. First, the study used data from a cross-sectional survey which makes it difficult to measure causality. To overcome this limitation, causal inference statistical methodologies was used to estimate average treatment effects of the interventions. The analysis adjusted for the treatment assignment with important variables in estimating potential outcomes of women whose households had the intervention should they not have the intervention as well as those whose households did not have the intervention should they have.

Secondly, access to ITNs does not necessarily imply utilization of ITNs, therefore, care must be taken in the interpretation of results and conclusions from this study. Thirdly, the outcome for this study self-reported malaria was a proxy to malaria prevalence among women in the past 12 months, hence could be biased by the knowledge level of the women on malaria, especially the unconfirmed positive cases.

Finally, the study did not account for multiple episodes of malaria cases per participants within the 1-year reference period as well as the exact timing of having the malaria episode and the interventions. Given the limitations of the observational study, a more robust randomized controlled trial would be an important consideration for future research study.

## Conclusion

Households with access to both ITNs and IRS had a lower prevalence of self-reported malaria compared to households with none of the two interventions. This finding confirms the call for integrating malaria control interventions to facilitate attainment of malaria elimination in Ghana.

## Supplementary Information

**Additional file 1: Table S1. **Sub analysis of the impact of malaria intervention on self-reported malaria prevalence among women by household and women characteristics.

## Data Availability

The GMIS data is available online at no cost at the DHS portal. It can be access through the website https://dhsprogram.com/data/ upon request [26].

## Abbreviations

DHS: Demographic health survey
GMIS: Ghana malaria indicator survey
IRS: Indoor residual spraying
ITNs: Insecticide-treated nets
LLINs: Long-lasting insecticidal nets
MIS: Malaria indicator survey
NMCP: National Malaria Control Programme
WHO: World Health Organization
