# Supplementary material for: Impact of insecticide-treated nets and indoor residual spraying on self-reported malaria prevalence among women of reproductive age in Ghana: implication for malaria control and elimination
Source: Malar J. 2022 Apr 12;21:120. doi: 10.1186/s12936-022-04136-3 (PMC9003985; doi:10.1186/s12936-022-04136-3)
Supplement: Supplementary file 1 — Additional file 1: Table S1. Sub analysis of the impact of malaria intervention on self-reported malaria prevalence among women by household and women characteristics. [file 12936_2022_4136_MOESM1_ESM.docx]

**Additional file 1: Table S1. Sub analysis of the impact of malaria intervention on self-reported malaria prevalence among women by household and women characteristics**

|  | **Access to ITNs vs. No access to ITNs** | |  | **Household sprayed (IRS) vs. Household not sprayed** | |  | **Access to ITNs & IRS vs. Access to ITNs only** | |  | **Access to ITNs & IRS vs. Access to IRS only** | |  | **Access to ITNs & IRS vs. No access to ITNs & IRS** | |
| --- | --- | --- | --- | --- | --- | --- | --- | --- | --- | --- | --- | --- | --- | --- |
| **Variables** | **ATE [95% CI]** | **P-value** |  | **ATE [95% CI]** | **P-value** |  | **ATE [95% CI]** | **P-value** |  | **ATE [95% CI]** | **P-value** |  | **ATE [95% CI]** | **P-value** |
| **Region of residence** |  |  |  |  |  |  |  |  |  |  |  |  |  |  |
| *Western* | -7.62 [-15.80, 0.56] | 0.068 |  | -4.52 [-10.36, 1.32] | 0.129 |  | -3.66 [-10.54, 3.23] | 0.296 |  | -1.66 [-7.29, 3.97] | 0.556 |  | -10.82 [-25.94, 4.29] | 0.159 |
| *Central* | -8.71 [-16.49, -0.92] | 0.029 |  | -9.35 [-19.03, 0.34] | 0.058 |  | -7.89 [-19.42, 3.64] | 0.178 |  | -4.26 [-11.44, 2.92] | 0.238 |  | -25.77 [-49.52, -2.01] | 0.034 |
| *Greater Accra* | -6.46 [-11.14, -1.79] | 0.007 |  | -4.10 [-7.37, -0.83] | 0.014 |  | -3.56 [-7.71, 0.58] | 0.092 |  | 0.00 [0.00, 0.00] | 0.438 |  | -10.84 [-18.40, -3.28] | 0.005 |
| *Volta* | -6.33 [-10.51, -2.15] | 0.003 |  | -7.29 [-12.78, -1.81] | 0.009 |  | -6.65 [-13.61, 0.30] | 0.061 |  | - |  |  | -15.04 [-22.18, -7.90] | <0.001 |
| *Eastern* | -7.86 [-13.66, -2.07] | 0.008 |  | -8.20 [-14.89, -1.52] | 0.016 |  | -6.37 [-12.93, 0.19] | 0.057 |  | - |  |  | -23.54 [-35.43, -11.65] | <0.001 |
| *Ashanti* | -6.61 [-13.26, 0.04] | 0.051 |  | -8.73 [-17.82, 0.35] | 0.060 |  | -6.98 [-15.13, 1.17] | 0.093 |  | -12.11 [-58.89, 34.68] | 0.605 |  | -29.34 [-56.51, -2.18] | 0.034 |
| *Brong Ahafo* | -8.43 [-17.70, 0.84] | 0.074 |  | -11.50 [-25.87, 2.86] | 0.116 |  | -8.59 [-20.27, 3.09] | 0.149 |  | -58.63 [-398.47, 281.22] | 0.730 |  | -36.42 [-80.79, 7.94] | 0.107 |
| *Northern* | -9.57 [-22.18, 3.04] | 0.136 |  | -16.91 [-42.44, 8.61] | 0.193 |  | -12.60 [-33.24, 8.03] | 0.230 |  | -14.97 [-121.59, 91.66] | 0.779 |  | -76.09 [-198.99, 46.82] | 0.223 |
| *Upper East* | -11.29 [-28.58, 6.00] | 0.199 |  | -18.66 [-50.27, 12.95] | 0.246 |  | -15.45 [-43.87, 12.97] | 0.285 |  | -22.52 [-204.48, 159.44] | 0.804 |  | -95.31 [-274.84, 84.23] | 0.296 |
| *Upper West* | -5.17 [-13.86, 3.53] | 0.243 |  | -13.52 [-40.87, 13.82] | 0.331 |  | -11.31 [-35.74, 13.11] | 0.362 |  | -9.11 [-88.79, 70.56] | 0.819 |  | -68.67 [-221.35, 84.02] | 0.376 |
| **Residence** |  |  |  |  |  |  |  |  |  |  |  |  |  |  |
| *Urban* | -7.14 [-12.13, -2.14] | 0.005 |  | -8.35 [-14.96, -1.75] | 0.013 |  | -6.68 [-14.13, 0.77] | 0.078 |  | -4.19 [-9.07, 0.70] | 0.091 |  | -24.22 [-32.66, -15.78] | <0.001 |
| *Rural* | -7.88 [-13.60, -2.16] | 0.007 |  | -8.30 [-14.64, -1.96] | 0.011 |  | -7.25 [-14.52, 0.03] | 0.051 |  | -5.35 [-11.71, 1.01] | 0.097 |  | -30.94 [-39.66, -22.22] | <0.001 |
| **HOUSEHOLD CHARACTERISTICS** |  |  |  |  |  |  |  |  |  |  |  |  |  |  |
| **Household size** |  |  |  |  |  |  |  |  |  |  |  |  |  |  |
| *<4 members* | -7.66 [-13.22, -2.11] | 0.007 |  | -10.05 [-18.10, -2.01] | 0.015 |  | -8.36 [-17.41, 0.69] | 0.070 |  | -3.06 [-6.54, 0.43] | 0.084 |  | -29.54 [-38.91, -20.17] | <0.001 |
| *4-6 members* | -7.80 [-13.61, -1.99] | 0.009 |  | -7.64 [-13.49, -1.80] | 0.011 |  | -6.56 [-13.16, 0.05] | 0.052 |  | -5.80 [-12.38, 0.77] | 0.082 |  | -27.33 [-35.92, -18.74] | <0.001 |
| *7-9 members* | -6.63 [-10.98, -2.28] | 0.003 |  | -8.02 [-14.25, -1.80] | 0.012 |  | -6.79 [-14.32, 0.73] | 0.077 |  | -5.01 [-12.13, 2.10] | 0.163 |  | -25.94 [-37.12, -14.76] | <0.001 |
| *10+ members* | -6.69 [-11.18, -2.20] | 0.004 |  | -7.73 [-13.95, -1.52] | 0.015 |  | -6.72 [-14.05, 0.62] | 0.072 |  | -5.82 [-14.48, 2.84] | 0.183 |  | -28.39 [-40.14, -16.65] | <0.001 |
| **Sex of household head** |  |  |  |  |  |  |  |  |  |  |  |  |  |  |
| *Male* | -7.52 [-13.00, -2.04] | 0.007 |  | -8.17 [-14.62, -1.71] | 0.013 |  | -6.95 [-14.18, 0.27] | 0.059 |  | -5.49 [-11.64, 0.67] | 0.079 |  | -27.91 [-36.49, -19.34] | <0.001 |
| *Female* | -7.39 [-12.45, -2.32] | 0.005 |  | -8.62 [-15.18, -2.06] | 0.010 |  | -7.07 [-14.76, 0.63] | 0.072 |  | -2.70 [-7.43, 2.04] | 0.257 |  | -27.30 [-37.35, -17.25] | <0.001 |
| **Age of household head (mean ± SD)** |  |  |  |  |  |  |  |  |  |  |  |  |  |  |
| *<30* | -7.75 [-13.01, -2.48] | 0.004 |  | -9.10 [-16.37, -1.84] | 0.014 |  | -7.17 [-15.30, 0.96] | 0.084 |  | -5.74 [-15.44, 3.97] | 0.240 |  | -27.41 [-38.05, -16.76] | <0.001 |
| *30-49* | -7.28 [-12.61, -1.95] | 0.008 |  | -8.17 [-14.49, -1.85] | 0.012 |  | -7.11 [-14.39, 0.17] | 0.055 |  | -4.78 [-10.14, 0.57] | 0.079 |  | -27.18 [-35.86, -18.51] | <0.001 |
| *50-69* | -7.89 [-13.44, -2.34] | 0.006 |  | -8.96 [-15.90, -2.02] | 0.012 |  | -7.33 [-15.38, 0.72] | 0.074 |  | -5.48 [-11.78, 0.82] | 0.087 |  | -29.44 [-39.14, -19.74] | <0.001 |
| *>69* | -6.86 [-12.10, -1.62] | 0.011 |  | -5.97 [-12.07, 0.12] | 0.055 |  | -4.24 [-9.86, 1.38] | 0.139 |  | -4.26 [-10.59, 2.08] | 0.182 |  | -30.08 [-50.50, -9.66] | 0.004 |
| **Wealth index** |  |  |  |  |  |  |  |  |  |  |  |  |  |  |
| *Poor* | -7.26 [-13.04, -1.47] | 0.014 |  | -9.16 [-16.56, -1.76] | 0.016 |  | -7.50 [-15.66, 0.66] | 0.071 |  | -5.71 [-13.84, 2.42] | 0.164 |  | -32.70 [-47.51, -17.90] | <0.001 |
| *Middle* | -8.23 [-13.88, -2.58] | 0.005 |  | -9.72 [-17.42, -2.03] | 0.014 |  | -8.23 [-17.26, 0.81] | 0.074 |  | -6.76 [-13.94, 0.42] | 0.064 |  | -31.98 [-42.93, -21.03] | <0.001 |
| *Rich* | -7.25 [-12.22, -2.28] | 0.005 |  | -7.19 [-12.78, -1.60] | 0.012 |  | -5.97 [-12.17, 0.24] | 0.059 |  | -2.44 [-5.01, 0.13] | 0.062 |  | -22.29 [-29.73, -14.85] | <0.001 |
| **Source of water** |  |  |  |  |  |  |  |  |  |  |  |  |  |  |
| *Improved water source* | -7.70 [-13.18, -2.21] | 0.006 |  | -8.50 [-15.09, -1.91] | 0.012 |  | -7.15 [-14.70, 0.39] | 0.063 |  | -5.35 [-11.94, 1.25] | 0.109 |  | -28.21 [-36.47, -19.96] | <0.001 |
| *Unimproved water source* | -5.80 [-10.12, -1.49] | 0.009 |  | -7.12 [-12.46, -1.79] | 0.009 |  | -6.04 [-12.15, 0.07] | 0.053 |  | -2.90 [-5.51, -0.29] | 0.030 |  | -23.17 [-33.36, -12.98] | <0.001 |
| **Toilet facility** |  |  |  |  |  |  |  |  |  |  |  |  |  |  |
| *Improved toilet facility* | -7.36 [-12.63, -2.10] | 0.006 |  | -9.17 [-16.20, -2.14] | 0.011 |  | -7.81 [-15.97, 0.35] | 0.060 |  | -5.46 [-12.22, 1.30] | 0.111 |  | -31.51 [-40.97, -22.04] | <0.001 |
| *Unimproved toilet facility* | -7.75 [-13.35, -2.16] | 0.007 |  | -6.92 [-12.46, -1.37] | 0.015 |  | -5.86 [-12.17, 0.45] | 0.068 |  | -4.07 [-8.33, 0.19] | 0.060 |  | -21.58 [-29.68, -13.49] | <0.001 |
| **Access to electricity** |  |  |  |  |  |  |  |  |  |  |  |  |  |  |
| *No* | -7.06 [-11.90, -2.22] | 0.004 |  | -10.79 [-19.27, -2.31] | 0.013 |  | -9.50 [-19.83, 0.83] | 0.071 |  | -8.29 [-19.95, 3.36] | 0.158 |  | -36.96 [-52.52, -21.40] | <0.001 |
| *Yes* | -7.56 [-13.02, -2.10] | 0.007 |  | -7.60 [-13.48, -1.71] | 0.012 |  | -6.13 [-12.49, 0.23] | 0.059 |  | -4.69 [-9.90, 0.52] | 0.076 |  | -26.26 [-34.26, -18.25] | <0.001 |
| **Main floor materials** |  |  |  |  |  |  |  |  |  |  |  |  |  |  |
| *Ceramic/tiles/carpet* | -7.40 [-12.63, -2.17] | 0.006 |  | -8.18 [-14.63, -1.72] | 0.013 |  | -7.78 [-15.42, -0.13] | 0.046 |  | -4.94 [-10.03, 0.15] | 0.057 |  | -32.13 [-43.22, -21.05] | <0.001 |
| *Cement* | -7.47 [-12.81, -2.14] | 0.006 |  | -8.40 [-14.87, -1.93] | 0.011 |  | -6.79 [-14.07, 0.49] | 0.067 |  | -4.84 [-10.50, 0.82] | 0.092 |  | -26.90 [-35.43, -18.37] | <0.001 |
| *Sand/earth/wooden planks* | -7.61 [-13.43, -1.79] | 0.011 |  | -8.16 [-14.73, -1.59] | 0.015 |  | -6.68 [-13.93, 0.56] | 0.070 |  | -8.02 [-19.26, 3.22] | 0.157 |  | -24.48 [-34.48, -14.48] | <0.001 |
| **Main wall materials** |  |  |  |  |  |  |  |  |  |  |  |  |  |  |
| *Cement/bricks* | -7.43 [-12.85, -2.02] | 0.007 |  | -8.46 [-14.97, -1.94] | 0.011 |  | -7.16 [-14.56, 0.24] | 0.058 |  | -7.61 [-16.93, 1.71] | 0.107 |  | -28.01 [-36.33, -19.70] | <0.001 |
| *Others (clay, woods etc.)* | -7.55 [-12.77, -2.33] | 0.005 |  | -8.11 [-14.46, -1.76] | 0.013 |  | -6.77 [-14.05, 0.51] | 0.068 |  | -4.09 [-8.86, 0.68] | 0.091 |  | -27.25 [-37.20, -17.29] | <0.001 |
| **Main roof materials** |  |  |  |  |  |  |  |  |  |  |  |  |  |  |
| *Asbestos/shingles/concrete* | -7.43 [-12.44, -2.42] | 0.004 |  | -6.53 [-11.09, -1.97] | 0.005 |  | -5.48 [-10.99, 0.03] | 0.051 |  | -2.09 [-6.94, 2.76] | 0.391 |  | -25.48 [-34.84, -16.11] | <0.001 |
| *Zinc/aluminium* | -7.56 [-13.03, -2.08] | 0.007 |  | -8.67 [-15.45, -1.90] | 0.012 |  | -7.24 [-14.87, 0.40] | 0.063 |  | -4.98 [-10.76, 0.80] | 0.090 |  | -28.27 [-36.85, -19.69] | <0.001 |
| *Thatch/palm leaves/wood* | -5.58 [-9.60, -1.55] | 0.007 |  | -7.23 [-13.57, -0.89] | 0.026 |  | -6.73 [-14.09, 0.64] | 0.073 |  | -9.54 [-22.06, 2.98] | 0.132 |  | -20.55 [-34.52, -6.58] | 0.004 |
| **Cooking fuel** |  |  |  |  |  |  |  |  |  |  |  |  |  |  |
| *Non-solid* | -7.62 [-12.87, -2.38] | 0.005 |  | -10.72 [-19.46, -1.98] | 0.017 |  | -8.06 [-17.13, 1.01] | 0.081 |  | -9.03 [-25.79, 7.72] | 0.283 |  | -31.18 [-41.72, -20.65] | <0.001 |
| *Solid* | -7.43 [-12.79, -2.07] | 0.007 |  | -7.76 [-13.71, -1.81] | 0.011 |  | -6.77 [-13.78, 0.23] | 0.058 |  | -4.91 [-10.44, 0.61] | 0.080 |  | -26.84 [-35.07, -18.62] | <0.001 |
| **WOMEN CHARACTERISTICS** |  |  |  |  |  |  |  |  |  |  |  |  |  |  |
| **Woman’s age** |  |  |  |  |  |  |  |  |  |  |  |  |  |  |
| *15-19* | -6.06 [-10.56, -1.56] | 0.009 |  | -7.07 [-13.60, -0.54] | 0.034 |  | -5.93 [-13.91, 2.05] | 0.144 |  | -7.21 [-18.07, 3.66] | 0.188 |  | -25.80 [-40.82, -10.79] | 0.001 |
| *20-29* | -7.67 [-13.11, -2.23] | 0.006 |  | -8.02 [-14.57, -1.47] | 0.017 |  | -7.29 [-15.07, 0.50] | 0.066 |  | -7.71 [-17.71, 2.29] | 0.128 |  | -25.92 [-34.56, -17.28] | <0.001 |
| *30-39* | -7.87 [-13.60, -2.14] | 0.007 |  | -8.76 [-15.27, -2.25] | 0.009 |  | -6.82 [-13.62, -0.03] | 0.049 |  | -2.89 [-6.83, 1.05] | 0.146 |  | -27.99 [-36.21, -19.77] | <0.001 |
| *40-49* | -7.66 [-13.13, -2.19] | 0.006 |  | -8.83 [-15.99, -1.68] | 0.016 |  | -7.56 [-15.45, 0.33] | 0.060 |  | -2.67 [-6.38, 1.05] | 0.155 |  | -31.54 [-42.85, -20.23] | <0.001 |
| **Woman’s education** |  |  |  |  |  |  |  |  |  |  |  |  |  |  |
| *No education* | -6.95 [-12.38, -1.52] | 0.012 |  | -7.83 [-14.03, -1.64] | 0.014 |  | -6.16 [-12.42, 0.10] | 0.054 |  | -5.20 [-11.51, 1.11] | 0.104 |  | -26.30 [-34.48, -18.12] | <0.001 |
| *Primary* | -6.97 [-11.60, -2.33] | 0.003 |  | -8.98 [-15.73, -2.23] | 0.009 |  | -7.38 [-14.99, 0.22] | 0.057 |  | -6.27 [-14.61, 2.08] | 0.137 |  | -25.79 [-35.13, -16.45] | <0.001 |
| *Secondary* | -7.79 [-13.34, -2.24] | 0.006 |  | -8.31 [-14.77, -1.85] | 0.012 |  | -7.16 [-14.71, 0.39] | 0.063 |  | -4.59 [-9.72, 0.54] | 0.078 |  | -28.10 [-37.25, -18.94] | <0.001 |
| *Higher / tertiary* | -7.84 [-13.66, -2.03] | 0.008 |  | -8.41 [-15.82, -1.00] | 0.026 |  | -7.55 [-17.79, 2.70] | 0.148 |  | - |  |  | -35.21 [-56.32, -14.10] | 0.001 |
| **Number of births** |  |  |  |  |  |  |  |  |  |  |  |  |  |  |
| *None* | -6.47 [-11.15, -1.78] | 0.007 |  | -6.67 [-12.83, -0.52] | 0.034 |  | -6.05 [-12.01, -0.08] | 0.047 |  | -0.89 [-2.31, 0.53] | 0.215 |  | -24.04 [-34.47, -13.61] | <0.001 |
| *1-2 births* | -7.27 [-12.36, -2.18] | 0.005 |  | -8.42 [-14.60, -2.23] | 0.008 |  | -6.33 [-12.89, 0.23] | 0.059 |  | -7.93 [-17.01, 1.14] | 0.085 |  | -25.71 [-33.43, -18.00] | <0.001 |
| *3-4 births* | -7.71 [-13.16, -2.27] | 0.006 |  | -7.79 [-13.94, -1.64] | 0.013 |  | -6.62 [-14.15, 0.91] | 0.085 |  | -6.16 [-13.48, 1.16] | 0.097 |  | -28.53 [-39.01, -18.04] | <0.001 |
| *>4 births* | -8.92 [-15.69, -2.15] | 0.010 |  | -10.38 [-19.00, -1.77] | 0.018 |  | -8.83 [-19.08, 1.42] | 0.091 |  | -8.60 [-21.93, 4.73] | 0.200 |  | -32.79 [-46.21, -19.38] | <0.001 |
| **Woman’s currently pregnant** |  |  |  |  |  |  |  |  |  |  |  |  |  |  |
| *No/unsure* | -7.54 [-12.93, -2.15] | 0.006 |  | -8.39 [-14.83, -1.95] | 0.011 |  | -7.04 [-14.39, 0.31] | 0.060 |  | -5.21 [-11.07, 0.64] | 0.080 |  | -27.43 [-35.38, -19.47] | <0.001 |
| *Yes* | -6.70 [-11.58, -1.83] | 0.007 |  | -7.36 [-13.45, -1.27] | 0.018 |  | -6.28 [-13.19, 0.62] | 0.074 |  | -2.70 [-8.00, 2.60] | 0.311 |  | -31.64 [-44.63, -18.65] | <0.001 |
| **Covered by health insurance** |  |  |  |  |  |  |  |  |  |  |  |  |  |  |
| *No* | -7.25 [-12.30, -2.20] | 0.005 |  | -8.17 [-14.48, -1.87] | 0.011 |  | -6.83 [-14.14, 0.49] | 0.067 |  | -5.39 [-11.70, 0.92] | 0.092 |  | -27.21 [-35.95, -18.47] | <0.001 |
| *Yes* | -7.62 [-13.14, -2.10] | 0.007 |  | -8.43 [-14.93, -1.93] | 0.011 |  | -7.11 [-14.43, 0.21] | 0.057 |  | -4.84 [-10.54, 0.86] | 0.094 |  | -28.02 [-36.34, -19.71] | <0.001 |
| **Woman’s religion** |  |  |  |  |  |  |  |  |  |  |  |  |  |  |
| *Christians* | -7.26 [-12.53, -2.00] | 0.007 |  | -8.13 [-14.34, -1.93] | 0.010 |  | -6.83 [-13.95, 0.29] | 0.060 |  | -4.16 [-8.84, 0.52] | 0.080 |  | -26.73 [-35.07, -18.38] | <0.001 |
| *Islam* | -8.47 [-14.18, -2.75] | 0.004 |  | -9.47 [-17.05, -1.88] | 0.015 |  | -7.74 [-16.04, 0.55] | 0.067 |  | -6.33 [-14.48, 1.81] | 0.124 |  | -30.11 [-40.43, -19.80] | <0.001 |
| *Tradition/No religion/ others* | -5.83 [-10.40, -1.26] | 0.013 |  | -5.68 [-11.73, 0.38] | 0.066 |  | -6.23 [-12.71, 0.25] | 0.060 |  | -2.35 [-8.66, 3.95] | 0.456 |  | -17.03 [-26.67, -7.38] | 0.001 |
| **Knowledge of malaria** |  |  |  |  |  |  |  |  |  |  |  |  |  |  |
| *Low knowledge* | -2.67 [-5.33, -0.02] | 0.048 |  | -1.90 [-3.95, 0.15] | 0.069 |  | -2.24 [-5.23, 0.74] | 0.139 |  | -1.74 [-7.42, 3.95] | 0.541 |  | -11.69 [-21.42, -1.96] | 0.019 |
| *Moderate knowledge* | -7.02 [-12.04, -2.00] | 0.006 |  | -7.89 [-13.78, -1.99] | 0.009 |  | -6.70 [-13.89, 0.50] | 0.068 |  | -3.03 [-6.89, 0.83] | 0.121 |  | -23.95 [-32.28, -15.61] | <0.001 |
| *Comprehensive knowledge* | -7.77 [-13.32, -2.22] | 0.006 |  | -8.68 [-15.49, -1.88] | 0.013 |  | -7.20 [-14.67, 0.28] | 0.059 |  | -5.43 [-11.86, 1.00] | 0.096 |  | -29.55 [-38.65, -20.45] | <0.001 |
| **Exposure to malaria messages in the past 6 months** |  |  |  |  |  |  |  |  |  |  |  |  |  |  |
| *Not exposed* | -6.89 [-11.88, -1.90] | 0.007 |  | -6.97 [-12.33, -1.60] | 0.011 |  | -5.74 [-11.71, 0.22] | 0.059 |  | -4.74 [-9.71, 0.24] | 0.062 |  | -24.22 [-32.32, -16.13] | <0.001 |
| *Exposed* | -8.14 [-13.90, -2.39] | 0.006 |  | -9.78 [-17.39, -2.17] | 0.012 |  | -8.26 [-17.05, 0.53] | 0.065 |  | -6.14 [-14.99, 2.72] | 0.170 |  | -33.14 [-42.55, -23.73] | <0.001 |
